# Can convergence in mixed-species flocks lead to evolutionary divergence? Evidence for and methods to test this hypothesis

**DOI:** 10.1098/rstb.2022.0112

**Published:** 2023-06-05

**Authors:** Rebecca T. Kimball, Edward L. Braun, Yang Liu, Liping Zhou, Eben Goodale, Wenyi Zhou, Scott K. Robinson

**Affiliations:** ^1^ Department of Biology, University of Florida, Gainesville, FL 32611, USA; ^2^ Florida Museum of Natural History, University of Florida, Gainesville, FL 32611, USA; ^3^ State Key Laboratory of Biocontrol, School of Ecology, Sun Yat-sen University, Guangzhou, Guangdong 510006, People's Republic of China; ^4^ Kunming Natural History Museum of Zoology, Kunming Institute of Zoology, Chinese Academy of Sciences, Kunming, Yunnan 650223, People's Republic of China; ^5^ Department of Health and Environmental Science, Xi'an Jiaotong-Liverpool University, Suzhou, Jiangsu 215123, People's Republic of China

**Keywords:** mimicry, convergent evolution, phenotypic matching, mixed-species flocks, speciation, incomplete-lineage sorting

## Abstract

One of the most fundamental goals of modern biology is to achieve a deep understanding of the origin and maintenance of biodiversity. It has been observed that in some mixed-species animal societies, there appears to be a drive towards some degree of phenotypic trait matching, such as similar coloration or patterning. Here we build on these observations and hypothesize that selection in mixed-species animal societies, such as mixed-species bird flocks, may drive diversification, potentially leading to speciation. We review evidence for possible convergent evolution and even outright mimicry in flocks from southwestern China, where we have observed several cases in which species and subspecies differ from their closest relatives in traits that match particular flock types. However, understanding whether this is phenotypic matching driven by convergence, and whether this divergence has promoted biodiversity, requires testing multiple facets of this hypothesis. We propose a series of steps that can be used to tease apart alternative hypotheses to build our understanding of the potential role of convergence in diversification in participants of mixed-species societies. Even if our social convergence/divergence hypothesis is not supported, the testing at each step should help highlight alternative processes that may affect mixed-species flocks, trait evolution and possible convergence.

This article is part of the theme issue ‘Mixed-species groups and aggregations: shaping ecological and behavioural patterns and processes’.

## 1. Introduction

Convergent evolution provides strong evidence for natural selection, especially among taxa spread far across the world that have evolved similar phenotypes in response to similar environmental selection pressures [1,2]. Recent applications of molecular phylogenies and comparative genomics have shown that convergent evolution is far more widespread than originally believed [3,4]. Convergent evolution is typically thought to reflect the limited set of dimensions available to exploit resources. For example, in birds, there has been repeated evolution of the same suite of traits related to foraging ecology, with many trait combinations not evolving [5]. Ultimately, this limits the diversity of biological forms.

The set of genomic mechanisms available to generate phenotypes may further limit phenotypic diversity. These genomic mechanisms represent a type of ‘internal constraint’ [6,7] that limits the ways organisms can undergo convergence. The existence of genomic constraints would predict that the evolution of similar phenotypes in disparate taxa typically involves mutations in the same gene (or small sets of genes). This has been called ‘biased convergence’ by Agrawal [4], and it represents a limitation on the amount of phenotypic space that organisms are able to exploit over evolutionary time. The relative contributions of selection due to the environment and internal constraints to convergent evolution are unclear, but it is clear that both phenomena limit biodiversity from a phenotypic perspective.

Perhaps paradoxically, convergence also has the potential to increase biodiversity via diversification and, eventually, speciation. For example, repeated movement of ancestral lineages into similar environments can yield ‘replicated’ sets of taxa with similar phenotypes [3]. Alternatively, mimics can converge on their models in Batesian or Muellerian systems [8,9] and, as a result of this convergence, diverge from their sister taxa. Phenotypic convergence has the potential to promote the coexistence of subordinate species with their models [10,11] by enabling subordinate species to essentially trick dominant species into allowing them to share resources from which they might otherwise be excluded. Finally, in mixed-species animal societies, there appears to be an evolutionary drive towards phenotypic similarity [12], potentially leading to social mimicry [13–15], the independent evolution of the same social signal among interacting species. The last of these contexts for convergence—mixed-species societies—could involve relatively subtle features that arise repeatedly. These subtle and, in some cases partial, similarities owing to convergence may easily be overlooked and thus may be more common than is currently recognized.

In this paper, we develop the hypothesis that convergent evolution, here focusing on the context of mixed-species societies, generates biodiversity by promoting evolutionary divergence within lineages. This flips dogma, which suggests contact between sympatric species leads to divergence and maintenance of existing species through competition and the avoidance of inbreeding or competition, the essence of character displacement [16,17]. Instead, we propose that in some contexts, mutualistic and commensal interactions between sympatric species may lead to reverse character displacement: sympatric species evolve similar rather than divergent phenotypes to facilitate co-existence in mixed-species societies—and that this may promote diversification, and ultimately speciation. Specifically, if multiple mixed-species systems occur within the range of a species, different populations of the species could evolve to match the phenotypes of the local mixed-species society. This localized trait matching will lead to phenotypic divergence from other populations of the same species (sub-speciation), producing incipient species (or subspecies). Here, we explore this hypothesis using mixed-species flocks of birds (MSFs), which are well-studied mixed-species societies that should be good model systems to explore the potential for this type of phenomenon

## 2. Plumage similarities in mixed-species flocks

The first step towards understanding the potential for convergence in MSFs to drive divergence is to assess whether flock members do share phenotypic similarities to a greater extent than would be expected by chance (given the community). Although competition can structure some MSF communities (e.g. in checkerboard distributions among close congeners [18,19]), the majority of evidence points towards facilitation as a predominant force: species tend to interact with other species that are similar, at least in terms of diet and body size [20–22]. Beyond diet and body size, however, an observation repeated over the past 50 years and across the world is that species in MSFs often resemble each other in plumage [13,14]. Resemblances in plumage have been repeatedly suggested among members of passerine-dominated MSFs [23–29], as well as in MSFs of shorebirds [30] and parrot aggregations [31].

There are multiple potential mechanisms for such resemblances. Similarities might facilitate communication [14]; this communication could be mutualistic, or even manipulative as is the case in vocal mimicry in MSFs [32,33], or similarities could lower the risk of a predator focusing on one individual (the ‘oddity effect’ [15,34]). Plumage mimicry in birds has been attributed to interspecific social dominance mimicry, in which a subordinate bird's similarities to a dominant one reduces aggression towards it [10,11,35], and this could allow species to forage in greater proximity in MSFs than they would otherwise be able to. Another reason a smaller species could imitate a larger one is that the larger bird may be a formidable adversary to a predator, which is consistent with recent studies of mimicry among woodpeckers [36,37]. Finally, one resemblance among Pitohui species (Family: Pachycephalidae) in MSFs of Papua New Guinea appears to be because a model species is toxic [38]. Importantly, resemblances in MSFs need not be complete, but can come at various levels of shared traits, such as wingbars, crests or belly colour [29].

To demonstrate plumage similarities in MSFs, we highlight flock systems of southwestern China, particularly in two Nature Reserves in Yunnan Province. As part of our work documenting basic MSF types in China (see the electronic supplementary material), we observed remarkable similarities between species in many MSFs. This led us to develop the ideas presented here about a role for convergent evolution in some MSF systems. Flocking is widespread in China, though we are only beginning to rigorously examine the issue of phenotypic similarities within these MSFs (e.g. Zhou *et al*. [29]). Using the online database ‘Birds of the World’ [39], we found 54% of the 652 passerine species of China have been described as associating in MSFs to at least some degree. In areas such as Ailaoshan National Nature Reserve in Yunnan Province, over 60% of individuals in a community can be found in MSFs at any one time [40]. Recent molecular phylogenies have begun to highlight high levels of phenotypic convergence in this region. For example, there are 14 species of ‘fulvettas’ in Yunnan that used to be assigned to the genus *Alcippe*, nearly all of which are core members of MSFs. These ‘fulvettas’ are now known to consist of at least four genera (i.e. *Schoeniparus*, *Alcippe*, *Fulvetta* and *Lioparus*) from three families (Pellorneidae, Alcippeidae and Paradoxornithidae). The polyphyly of the ‘fulvettas’ suggests that convergence of distantly related taxa may be quite common [41]. Below, we report observations by the coauthors in Yunnan and, to a lesser extent, Sichuan, from 2012 to 2022 that highlight cases where we feel MSFs exhibit plumage similarities.

**Figure 1.**
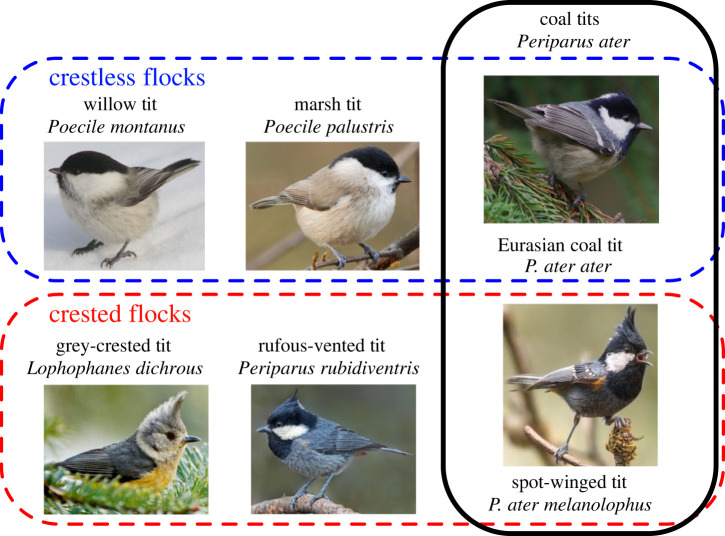
The presence or absence of crests in the coal tit (*Periparus ater*) complex depends on the presence or absence of crests in other flock members. Although subspecies of the coal tit differ in the presence of a crest, the crested/crestless phenotype correlates with flock members. See the electronic supplementary material for sources of the photographs. (Online version in colour.)

Convergence may be at the subspecies or species level and may take several forms. In some, convergence may occur among relatives (within the same genus or family), but not putative sister taxa. For example, one subspecies of coal tit (*Periparus ater melanolophus*) exhibits crests and participates in MSFs with other tit species that have crests in the Himalayas and Hengduan Mountains of southwestern China, whereas another (*Periparus ater ater*) lacks crests and flocks with other tits that lack crests in much of eastern China (figure 1).

**Figure 2.**
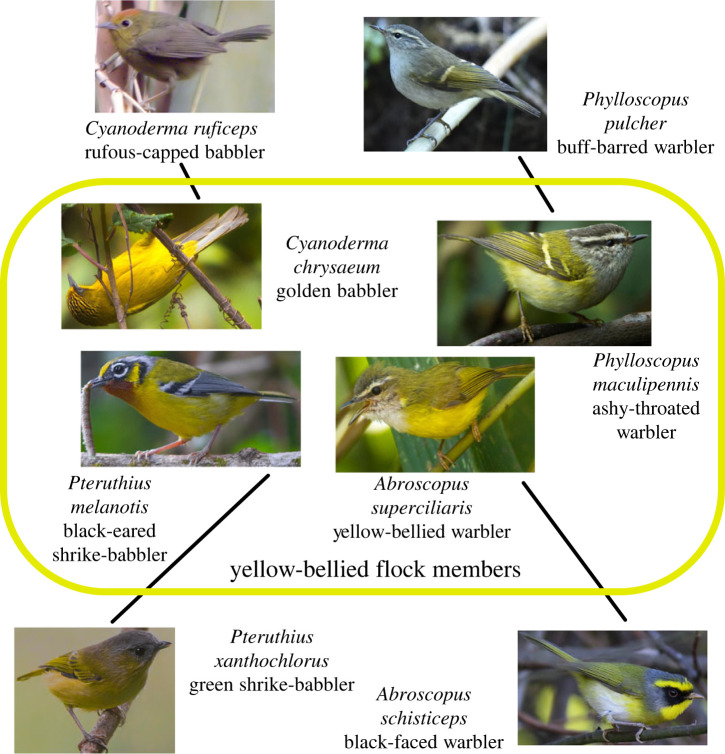
Convergence of yellow-bellied flock system. These taxa often have close relatives without a yellow belly (taxa outside the yellow belly box). The yellow sides of *Phylloscopus pulcher* are very different from the more intense yellow-coloured belly of *Phylloscopus maculipennis.* See the electronic supplementary material for sources of the photographs. (Online version in colour.)

In other cases, MSFs may be complex groups of diverse taxa, but still share key phenotypic traits. For example, we have sometimes (but not always) observed that different species with yellow bellies associate together in their own MSF or within larger MSFs that include species without yellow bellies. This is a taxonomically diverse system—40 species from 16 families and 23 genera—with yellow underparts. In some cases, the putative sister taxa for some of the participating species do not have this trait (figure 2).

There are several other examples of shared traits in MSFs of southwestern China. Subspecies of white-crested laughingthrush (*Garrulax leucolophus leucolophus* and *Garrulax leucolophus diardi*) and several scimitar-babblers (*Pomatorhinus ferruginosus, Pomatorhinus phayrei* and *Pomatorhinus ochraceiceps*) have rusty underparts in the Himalayas, where they flock with other species having rufous coloured bellies. However, these subspecies have white underparts further south in Yunnan and Myanmar, where they flock with a wide diversity of other white-bellied species. Similarly, several leaf warbler species (e.g. *Phylloscopus castaniceps, Phylloscopus forresti* and *Phylloscopus pulcher*) regularly flock with the rufous-faced warbler (*Abroscopus albogularis*). All species in this MSF system exhibit bright rumps that are most visible during hovering flight (electronic supplementary material, figure S1), which these species deploy frequently when foraging.

In addition, MSF members may share strikingly similar overall plumage patterns. For example, we have observed consistent co-occurrence of the Himalayan cutia (*Cutia nipalensis*) and rufous-backed sibia (*Leioptila annectens*), both in the family Leiothrichidae, in the same MSF (electronic supplementary material, figure S2). These two species have similar patterning with black heads, chestnut backs with dark wings and light underparts, and also have similar foraging behaviours: both are slow-moving foragers that search for insects along trunks and branches. On at least one occasion, we also observed the similarly plumaged black-headed shrike-babbler (Vireonidae: *Pteruthius rufiventer*) foraging with these two species.

**Figure 3.**
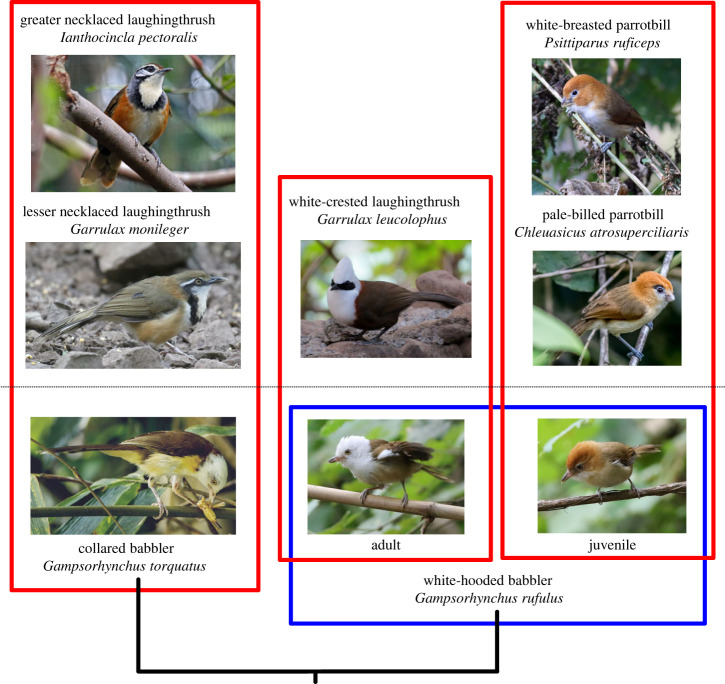
Complex convergence with flock members in white-hooded and collared babblers. These babbler species were considered conspecifics until recently and both share some phenotypic traits with laughingthrushes, with which they co-occur in MSFs. Juvenile white-hooded babblers flock with parrotbills and resemble those taxa phenotypically. See the electronic supplementary material for sources of the photographs. (Online version in colour.)

There are other cases of species that both flock together and were considered closely related owing to a high degree of phenotypic similarity. However, recent studies have demonstrated the taxa are more divergent and thus the phenotypic similarity is probably owing to convergence. For example, the greater (*Ianthocincla pectoralis*) and lesser necklaced (*Garrulax monileger*) laughingthrushes share many similarities, including the necklace (figure 3, left side). These two species associate closely in MSFs from Nepal to northeastern India, Myanmar, southwest China and even southeastern China (e.g. Shen *et al*. [42]). Once thought to be close relatives, they are now thought to be in different genera [43].

Flocking with the greater and lesser necklaced laughingthrushes is the collared babbler (*Gampsorhynchus torquatus*) (figure 3). The collared and white-hooded babblers (*Gampsorhynchus rufulus*) used to be considered conspecific, but are now separate species [44]. Our field observations have shown that the white-hooded babbler flocks with white-crested laughingthrushes in western Yunnan, which they resemble (figure 3). In the same MSF there are parrotbills, which themselves resemble juvenile white-hooded babblers. The two similar-looking parrotbills, white-breasted (*Psittiparus ruficeps*) and pale-billed (*Chleuasicus atrosuperciliaris*) parrotbills, forage side-by-side within these MSFs. They were thought to be sister species but are now placed in separate genera [45]. Thus, this system exemplifies multiple cases of likely MSF-driven convergence.

Given these observations, it is clear that MSFs comprise a wide variety of taxonomically distinct species, and that different MSF systems with unique phenotypes occur, even within the same region. Within species, different subspecies may participate in different MSF systems in different regions. We feel this leads to the possibility that convergence may drive divergence within species.

## 3. Testing for convergence owing to phenotypic matching in mixed-species flocks

**Figure 4.**
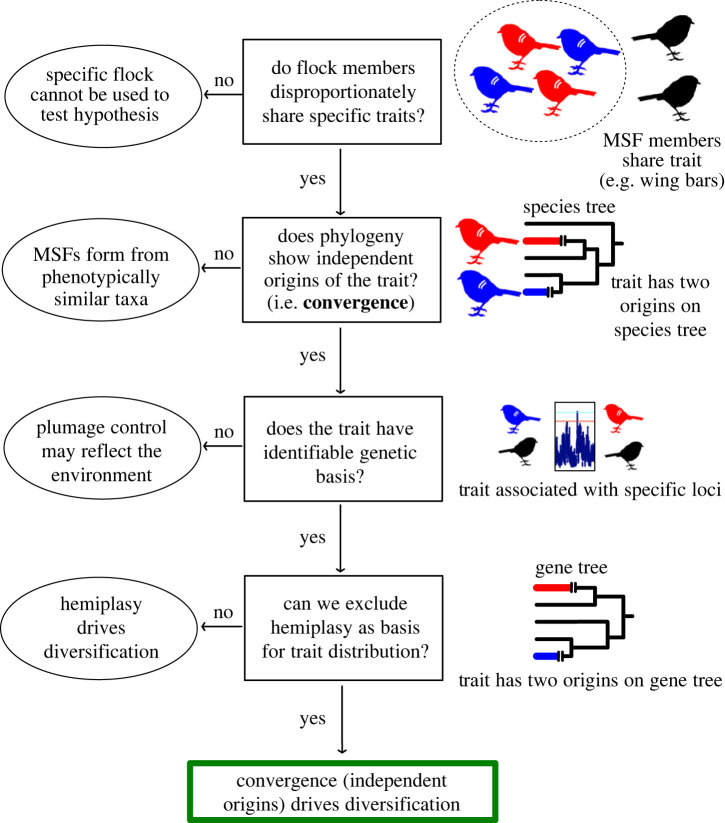
Criteria to determine whether evolutionary divergence is driven by phenotypic convergence, using MSFs as an example. (i) Do taxa in the same MSF share a phenotypic trait (red and blue birds with wing bars) not shared by taxa outside the MSF (black birds) in the same environment? (ii) Does the best estimate of phylogeny (a ‘species’ tree) show multiple origins of the trait? (iii) Does the trait have a genetic basis based on comparative genomic identification of alleles that correlate with the trait (e.g. *F*_st_ outlier analysis)? (iv) Integrating phylogeny and genetics, does the allele correlated with the trait show independent origins on its gene tree? If answers to all these questions are yes, then convergence owing to flocking may drive divergence. (Online version in colour.)

Assessing whether convergent evolution in MSFs represents an underappreciated (and currently understudied) mechanism to promote biodiversity requires an approach integrating knowledge of phylogeny, genetics and the composition of mixed-species associations across the range of focal species. To demonstrate that similarities between species result from convergent evolution owing to their social environment (i.e. membership in an MSF), we identified four steps (figure 4) that we feel must be applied to a specific mixed-species group. To support the overall hypothesis (convergence owing to social environment drives divergence) requires data that support a positive (‘yes’) response to the question at each step; at any step if data lead to a ‘no’ answer, the hypothesis is refuted.

First, the social convergence/divergence hypothesis requires repeated associations between a specific trait and MSF membership. In essence, species that participate in MSFs should share a specific phenotypic trait to a greater degree than occurs in the community as a whole. If not (i.e. MSF members are no more similar to each other than to other members of the community for a specific trait), then MSF membership is probably independent of phenotype. While assessing this is challenging, identification of species that are participating in MSFs, as well as species occupying the same habitat that are not participating in MSFs, will provide data to address this (see [29] for a quantitative examination of phenotypic matching, including plumage traits, at Tongbiguang Nature Reserve, Yunnan in southwestern China). Even more detailed observations can determine whether within microhabitats (e.g. forest understorey) traits are shared more within MSF participants than with the other species in the microhabitat, and further whether species with similar traits stay near to each other in MSFs [38]. Traits to be investigated include both overall colour tones, specific plumage characteristics such as belly colour or wingbars, or crests. In some cases, there may be species that participate rarely or occasionally, but with careful fieldwork it should be possible to identify common participants in MSFs (where phenotypic matching might be expected) versus sporadic participants (where there should not be selection for phenotypic matching). Examination of birds in the hand or museum skins may then allow assessment of the phenotypes of species that can be incorporated into analyses by allowing more careful assessment of traits (e.g. colour patches or features that may not always be visible in the field) as well as spectrophotometric analyses. Other processes can also lead to phenotypic similarities among co-occurring species, such as adaptation to specific environments. However, in these cases species that participate in an MSF and those that do not are sharing the same habitat/microhabitat and so should be equally similar and thus would lead to answering ‘no’ to step (i).

Second, the trait must have arisen independently based on the species tree of the entire MSF community. Species trees reflect the underlying patterns of diversification for the organisms rather than the relationships for any individual genetic locus, so their estimation requires the sequences of many unlinked genes. The social convergence/divergence hypothesis predicts that members of the MSF gained the trait independently from all or most other MSF members; even if two species in an MSF share the trait owing to a single origin, as long as many other MSF members gained the trait independently, this would support the prediction. If MSF members share a trait owing to a shared evolutionary history (single gain of the trait, even if some species may have subsequently lost the trait), then convergence is not leading to similarities among MSF members.

Third, the social convergence/divergence hypothesis predicts that the phenotypic differences have a genetic, not an environmental basis. While a genetic basis for many phenotypic differences is often assumed, it is clear that some phenotypic differences are environmentally induced or mediated. Indeed, phenotypic plasticity for the types of traits often assumed to delineate species or subspecies has been found [46], although it is thought to be rare in birds [47]. Genome comparisons can identify the genes underlying the convergence (e.g. through genomic scan analysis). Convergent evolution may be owing to either: (i) mutations in the same gene across many taxa (biased convergence [4]), or (ii) from mutations in different loci or regions of the genome for each taxon exhibiting a trait.

Fourth, if convergence explains the evolution of matching traits, then candidate gene(s) associated with the phenotype of interest should not represent discordant gene tree(s) consistent with a single origin of the trait, which would suggest that incomplete lineage sorting explains the presence of the trait [48]. Even if the species tree suggests a trait was gained independently, the alleles that affect the phenotype may have been gained a single time and are present in disparate species owing to incomplete lineage sorting, which can allow persistence of an allele over long time periods [49]. In other words, alleles responsible for an ancestral polymorphism for the trait (e.g. presence versus absence of a wing bar) might have been passed down to descendent species in a way that does not track directly with speciation patterns.

Cases where the sorting of alleles into descendent taxa results in the appearance of convergence are called hemiplasy [50], and they stand in contrast with homoplasy (true independent origins of a character). The existence of apparent convergence owing to differences between the evolutionary history of genes and species may seem surprising, but a model recently developed by Guerrero & Hahn [48] suggests it can be extremely common. If we apply the Guerrero & Hahn [48] results with coalescent branch length estimates from a recent whole-genome phylogeny of *Gallus* [51], we come to a striking conclusion: the likelihood that a trait which appears homoplastic within *Gallus* actually reflects incomplete lineage sorting (i.e. hemiplasy) is at least 10 times higher than the probability that the trait is genuinely homoplastic (unless the rate of mutation to the novel phenotype is very high). This example emphasizes that gene tree-species tree discordance represents a reasonable explanation for the apparent convergence of some traits. It is possible to test for hemiplasy by examining the phylogenetic tree for an individual gene associated with the trait of interest (identified by comparative genomics) with the species tree, as shown in figure 4. In essence, if the gene tree for a gene of interest (e.g. a trait that affects wing bars) suggests a single origin of the trait (wing bars) in the group of interest, this would suggest hemiplasy rather than independent origins (even if the species tree suggested that wing bars may have evolved more than once).

Support for these steps (i.e. cases where there is evidence for convergence based on the species tree and the trait has a genetic basis—see rectangles in figure 4) represent cases where membership in a mixed-species society may be driving phenotypic divergence among closely related taxa. Of course, there is a continuum of what could be observed. MSF members may be distinct from their sister taxa (other populations, subspecies or species) in different MSF systems, but without genome-wide differentiation. Instead, differentiation could be restricted to one or a few loci. This might suggest recent MSF membership that, if the association persists, could lead to divergence (and potentially speciation). At the other extreme, sister taxa may show clear, genome-wide differentiation, as well as evidence of selection at candidate loci that might suggest phenotypic diversification occurred more distantly in the past.

Currently, there are no data for MSFs that fully support all four steps in figure 4. However, there are currently data (see above) that strongly support step (i) (members of MSFs are phenotypically similar) for some MSFs. Additionally, the members of these MSFs are taxonomically diverse and differ phenotypically from closely related sister taxa, supporting step (ii). While appropriate genome data do not yet exist for MSF members (and their sister taxa) to address questions (iii) and (iv), comparative genomic studies in many avian taxa suggest plumage coloration typically has a genetic basis [52–54], suggesting step (iii) is likely to be true for at least some species in MSFs that exhibit phenotypic similarities (i.e. loci of relevant function will be among the set of candidate loci). The large taxonomic differences among taxa in some MSFs (e.g. different families; see above) suggest that at least some cases may reflect true convergence (not incomplete lineage sorting), supporting step (iv). Thus, while there are not currently data that address all of these steps in the same MSF system, it is likely that convergence in MSFs for plumage similarities may drive diversification. Additionally, studies that test each of these steps, even if failing to support the social convergence/divergence hypothesis, should still yield insights into the processes governing dynamics of MSFs and trait evolution.

More globally, this approach could be used to test the extent to which convergence in many different contexts might drive diversification. Here we have used MSFs to outline approaches for exploring this question, but it remains unknown the extent to which MSFs (or other interspecific social groupings) may drive convergence and ultimately diversification. In addition, apparent convergence may, in some cases, be owing to other factors. For example, predation may drive some species to participate in MSFs and concurrently there may be selection for phenotypes that minimize predation, leading to non-random patterns of phenotypic similarities in MSFs. Careful consideration of potential alternative explanations, which may be unique to different study systems, should also be considered.

## Data Availability

The data are provided in the electronic supplementary material [55].

## References

[1] Simpson GG. 1953 The major features of evolution. New York, NY: Columbia University Press.

[2] Wake DB, Wake MH, Specht CD. 2011 Homoplasy: from detecting pattern to determining process and mechanism of evolution. Science **331**, 1032-1035. (10.1126/science.1188545) 21350170

[3] Losos JB. 2011 Convergence, adaptation, and constraint. Evolution **65**, 1827-1840. (10.1111/j.1558-5646.2011.01289.x) 21729041

[4] Agrawal AA. 2017 Toward a predictive framework for convergent evolution: integrating natural history, genetic mechanisms, and consequences for the diversity of life. Am. Nat. **190**, S1-S12. (10.1086/692111) 28731831

[5] Pigot AL et al. 2020 Macroevolutionary convergence connects morphological form to ecological function in birds. Nat. Ecol. Evol. **4**, 230-239. (10.1038/s41559-019-1070-4) 31932703

[6] Wake DB. 1991 Homoplasy: the result of natural selection, or evidence of design limitations? Am. Nat. **138**, 543-567. (10.1086/285234)

[7] Schwenk K. 1995 A utilitarian approach to evolutionary constraint. Zoology **98**, 251-262.

[8] Wickler W. 1968 Mimicry in plants and animals. New York, NY: McGraw-Hill.

[9] Palmer DH, Kronforst MR. 2020 A shared genetic basis of mimicry across swallowtail butterflies points to ancestral co-option of doublesex. Nat. Commun. **11**, 6. (10.1038/s41467-019-13859-y) 31900419 PMC6941989

[10] Diamond JM. 1982 Mimicry of friarbirds by orioles. Auk **99**, 187-196.

[11] Prum RO. 2014 Interspecific social dominance mimicry in birds. Zool. J. Linn. Soc. **172**, 910-941. (10.1111/zoj.12192)

[12] Goodale E et al. 2020 Mixed company: a framework for understanding the composition and organization of mixed-species animal groups. Biol. Rev. Camb. Phil. Soc. **95**, 889-910. (10.1111/brv.12591) PMC7383667 32097520

[13] Beauchamp G, Goodale E. 2011 Plumage mimicry in avian mixed-species flocks: more or less than meets the eye? Auk **128**, 487-496. (10.1525/auk.2011.11016)

[14] Moynihan M. 1968 Social mimicry; character convergence versus character displacement. Evolution **22**, 315. (10.2307/2406531) 28564812

[15] Barnard CJ. 1979 Predation and the evolution of social mimicry in birds. Am. Nat. **113**, 613-618. (10.1086/283419)

[16] Lack D. 1983 Darwin‘s finches. Cambridge, UK: Cambridge University Press.

[17] Grant PR, Grant BR. 2006 Evolution of character displacement in Darwin's finches. Science **313**, 224-226. (10.1126/science.1128374) 16840700

[18] Graves GR, Gotelli NJ. 1993 Assembly of avian mixed-species flocks in Amazonia. Proc. Natl Acad. Sci. USA **90**, 1388-1391. (10.1073/pnas.90.4.1388) 8433996 PMC45878

[19] Colorado GJ, Rodewald AD. 2015 Assembly patterns of mixed-species avian flocks in the Andes. J. Anim. Ecol. **84**, 386-395. (10.1111/1365-2656.12300) 25283441

[20] Sridhar H et al. 2012 Positive relationships between association strength and phenotypic similarity characterize the assembly of mixed-species bird flocks worldwide. Am. Nat. **180**, 777-790. (10.1086/668012) 23149402

[21] Jones HH, Walters MJ, Robinson SK. 2020 Do similar foragers flock together? Nonbreeding foraging behavior and its impact on mixed-species flocking associations in a subtropical region. Auk **137**, ukz079. (10.1093/auk/ukz079)

[22] Mammides C, Chen J, Goodale UM, Kotagama SW, Goodale E. 2018 Measurement of species associations in mixed-species bird flocks across environmental and human disturbance gradients. Ecosphere **9**, e02324. (10.1002/ecs2.2324)

[23] Moynihan M. 1962 The organization and probable evolution of some mixed-species flocks of Neotropical birds. Smithsonian Misc. Collections **143**, 1-140.

[24] Moynihan M. 1979 Geographic variation in social behavior and in adaptations to competition among Andean birds. Publ. Nutall Ornithol. Club **18**, 1-162.

[25] Bell HL. 1983 A bird community of lowland rainforest in New Guinea. 5. Mixed-species feeding flocks. Emu - Austral Ornithol. **82**, 256-275. (10.1071/MU9820256s)

[26] Willis EO. 1989 Mimicry in bird flocks of cloud forests in southeastern Brazil. Revista Brasileira de Biol. **49**, 615-619.

[27] Sazima I. 2013 Five instances of bird mimicry suggested for Neotropical birds: a brief reappraisal. Revista Brasileira de Ornitol. **18**, 328-335.

[28] Goodale E, Goodale U, Mana R. 2012 The role of toxic pitohuis in mixed-species flocks of lowland forest in Papua New Guinea. Emu - Austral Ornithol. **112**, 9-16. (10.1071/MU11026)

[29] Zhou L et al. In press. High association strengths are linked to phenotypic similarity, including plumage color and patterns, of participants in mixed-species bird flocks of southwestern China. Curr. Zool. (10.1093/cz/zoac096) PMC10926261 38476134

[30] Cestari C. 2009 Heterospecific sociality of birds on beaches from southeastern Brazil. Zoologia (Curitiba, Impr.) **26**, 594-600. (10.1590/S1984-46702009005000013)

[31] Ferdinand V, Pattenden E, Brightsmith D, Hobson EA. 2023 Inferring the identifying features underlying association preferences in wild mixed species parrot groups. Phil. Trans. R. Soc. B **378**, 20220101. (10.1098/rstb.2022.0101) 37066652 PMC10107227

[32] Goodale E, Kotagama SW. 2006 Vocal mimicry by a passerine bird attracts other species involved in mixed-species flocks. Anim. Behav. **72**, 471-477. (10.1016/j.anbehav.2006.02.004)

[33] Flower TP, Gribble M, Ridley AR. 2014 Deception by flexible alarm mimicry in an African bird. Science **344**, 513-516. (10.1126/science.1249723) 24786078

[34] Jeschke JM, Tollrian R. 2007 Prey swarming: which predators become confused and why? Anim. Behav. **74**, 387-393. (10.1016/j.anbehav.2006.08.020)

[35] Jønsson KA, Delhey K, Sangster G, Ericson PGP, Irestedt M. 2016 The evolution of mimicry of friarbirds by orioles (Ayes: Passeriformes) in Australo-Pacific archipelagos. Proc. R. Soc. B **283**, 20161497. (10.1098/rspb.2016.1497) PMC4936029 27335418

[36] Miller ET, Leighton GM, Freeman BG, Lees AC, Ligon RA. 2019 Ecological and geographical overlap drive plumage evolution and mimicry in woodpeckers. Nat. Commun. **10**, 1602. (10.1038/s41467-019-09721-w) 30962513 PMC6453948

[37] Leighton GM, Lees AC, Miller ET. 2018 The hairy–downy game revisited: an empirical test of the interspecific social dominance mimicry hypothesis. Anim. Behav. **137**, 141-148. (10.1016/j.anbehav.2018.01.012)

[38] Diamond JM. 1992 Rubbish birds are poisonous. Nature **360**, 19-20. (10.1038/360019a0) 1436068

[39] Billerman SM, Keeney BK, Rodewald PG, Schulenberg TS. (eds). 2022 *Birds of the world*. Ithaca, NY: Cornell Laboratory of Ornithology. See https://birdsoftheworld.org/bow/home (accessed on 7 July 2022).

[40] Zhou L et al. 2019 The response of mixed-species bird flocks to anthropogenic disturbance and elevational variation in southwest China. Condor **121**, duz028. (10.1093/condor/duz028)

[41] Laiolo P, Seoane J, Obeso JR, Illera JC. 2017 Ecological divergence among young lineages favours sympatry, but convergence among old ones allows coexistence in syntopy. Glob. Ecol. Biogeogr. **26**, 601-608. (10.1111/geb.12565)

[42] Shen Y, Holyoak M, Goodale E, Mammides C, Zou F, Chen Y, Zhang C, Quan Q, Zhang Q. 2022 Mixed-species bird flocks re-assemble interspecific associations across an elevational gradient. Proc. R. Soc. B **289**, 20221840. (10.1098/rspb.2022.1840) PMC9768660 36541168

[43] Cai T et al. 2019 Near-complete phylogeny and taxonomic revision of the world's babblers (Aves: Passeriformes). Mol. Phylogenet. Evol. **130**, 346-356. (10.1016/j.ympev.2018.10.010) 30321696

[44] Collar NJ. 2006 A partial revision of the Asian babblers (Timaliidae). Forktail **22**, 85-112.

[45] Liu Y, Hu J, Li S-H, Duchen P, Wegmann D, Schweizer M. 2016 Sino-Himalayan mountains act as cradles of diversity and immigration centres in the diversification of parrotbills (Paradoxornithidae). J. Biogeogr. **43**, 1488-1501. (10.1111/jbi.12738)

[46] Vane-Wright RI, Tennent WJ. 2011 Colour and size variation in *Junonia villida* (Lepidoptera, Nymphalidae): subspecies or phenotypic plasticity? Syst. Biodivers. **9**, 289-305. (10.1080/14772000.2011.640497)

[47] Merilä J, Sheldon BC. 2001 Avian quantitative genetics. In Current ornithology, volume 16 (eds V Nolan, CF Thompson), pp. 179-255. Boston, MA: Springer US.

[48] Guerrero RF, Hahn MW. 2018 Quantifying the risk of hemiplasy in phylogenetic inference. Proc. Natl Acad. Sci. USA **115**, 12 787-12 792. (10.1073/pnas.1811268115) PMC6294915 30482861

[49] Hudson RR, Coyne JA. 2002 Mathematical consequences of the genealogical species concept. Evolution **56**, 1557-1565. (10.1111/j.0014-3820.2002.tb01467.x) 12353748

[50] Avise JC, Robinson TJ. 2008 Hemiplasy: a new term in the lexicon of phylogenetics. Syst. Biol. **57**, 503-507. (10.1080/10635150802164587) 18570042

[51] Tiley GP, Pandey A, Kimball RT, Braun EL, Burleigh JG. 2020 Whole genome phylogeny of *Gallus*: introgression and data-type effects. Avian Res. **11**, 7. (10.1186/s40657-020-00194-w)

[52] Toews DPL, Taylor SA, Vallender R, Brelsford A, Butcher BG, Messer PW, Lovette IJ. 2016 Plumage genes and little else distinguish the genomes of hybridizing warblers. Curr. Biol. **26**, 2313-2318. (10.1016/j.cub.2016.06.034) 27546575

[53] Stryjewski KF, Sorenson MD. 2017 Mosaic genome evolution in a recent and rapid avian radiation. Nat. Ecol. Evol. **1**, 1912-1922. (10.1038/s41559-017-0364-7) 29085063

[54] Aguillon SM, Walsh J, Lovette IJ. 2021 Extensive hybridization reveals multiple coloration genes underlying a complex plumage phenotype. Proc. R. Soc. B **288**, 20201805. (10.1098/rspb.2020.1805) PMC7893273 33468000

[55] Kimball RT, Braun EL, Liu Y, Zhou L, Goodale E, Zhou W, Robinson SK. 2023 Can convergence in mixed-species flocks lead to evolutionary divergence? Evidence for and methods to test this hypothesis. *Figshare*. (10.6084/m9.figshare.c.6461014) PMC10107229 37066651

